# Adult classical glioblastoma with a *BRAF* V600E mutation

**DOI:** 10.1186/s12957-015-0521-x

**Published:** 2015-03-11

**Authors:** Yoshinobu Takahashi, Toshiaki Akahane, Takahiro Sawada, Hidetoshi Ikeda, Akira Tempaku, Shigeru Yamauchi, Hiroshi Nishihara, Shinya Tanaka, Kazumi Nitta, Wataru Ide, Ikuo Hashimoto, Hajime Kamada

**Affiliations:** Department of Neurosurgery, Hokuto Hospital, 7-5, Inada, Obihiro, Hokkaido 080-0039 Japan; Department of Pathology, Hokuto Hospital, 7-5, Inada, Obihiro, Hokkaido 080-0039 Japan; Department of Biology and Genetics, Laboratory of Cancer Medical Science, Hokuto Hospital, 7-5, Inada, Obihiro, Hokkaido 080-0039 Japan; Department of Translational Pathology, Hokkaido University Graduate School of Medicine, N15, W7, Kita-ku, Sapporo, Hokkaido 060-8638 Japan

**Keywords:** *BRAF* V600E, Adult, Classical glioblastoma

## Abstract

The B-Raf proto-oncogene serine/threonine kinase (B-Raf) is a member of the Raf kinase family. The *BRAF* V600E mutation occurs frequently in certain brain tumors such as pleomorphic xanthoastrocytoma, ganglioglioma, and pilocytic astrocytoma, and less frequently in epithelioid and giant cell glioblastoma. *BRAF* V600E mutation in these cases has been canonically detected using Sanger sequencing or immunohistochemistry but not with next-generation sequencing (NGS). Moreover, to our knowledge, there is no detailed report of the *BRAF* V600E mutation in an adult glioblastoma with classical histologic features (c-GBM). Therefore, we performed NGS analysis to determine the mutational status of *BRAF* of 13 glioblastomas (GBMs) (11 primary and 2 secondary cases) and detected one tumor harboring the *BRAF* V600E mutation. We report here the detection of the *BRAF* V600E mutation in a patient with c-GBM and describe the patient’s clinical course as well as the results of histopathological analysis.

## Background

The B-Raf proto-oncogene serine/threonine kinase (B-Raf) is a strong activator of the extracellular signal-regulated kinase/mitogen-activated protein kinase 1 and 2 (Erk 1/2) signal transduction cascade that mediates cell proliferation [1]. Most *BRAF* mutations occur at codon 600, which resides within the activation loop of the kinase domain, and 80% to 90% of these mutations generate a protein with a glutamic acid (E) residue substituted for the normal valine (V) residue (*BRAF* V600E). Such mutant proteins exhibit increased kinase activity and transform cultured cells. The *BRAF* V600E mutation occurs frequently in certain brain tumors such as pleomorphic xanthoastrocytoma (PXA) (60%), PXA with anaplastic features (60%), ganglioglioma (20% to 60%), extracerebellar pilocytic astrocytoma (20%) [2-5], epithelioid glioblastoma (54%) [6], and giant cell glioblastoma (7%) [5]. However, the few studies of adult classical glioblastoma (c-GBM) with the *BRAF* V600E mutation lack detailed characterization of the tumors. Here, we present the first report, to our knowledge, that combines histopathological, immunohistochemical, and next-generation sequencing (NGS) analyses of c-GBM with the *BRAF* V600E mutation.

## Case presentation

**Figure 1:**
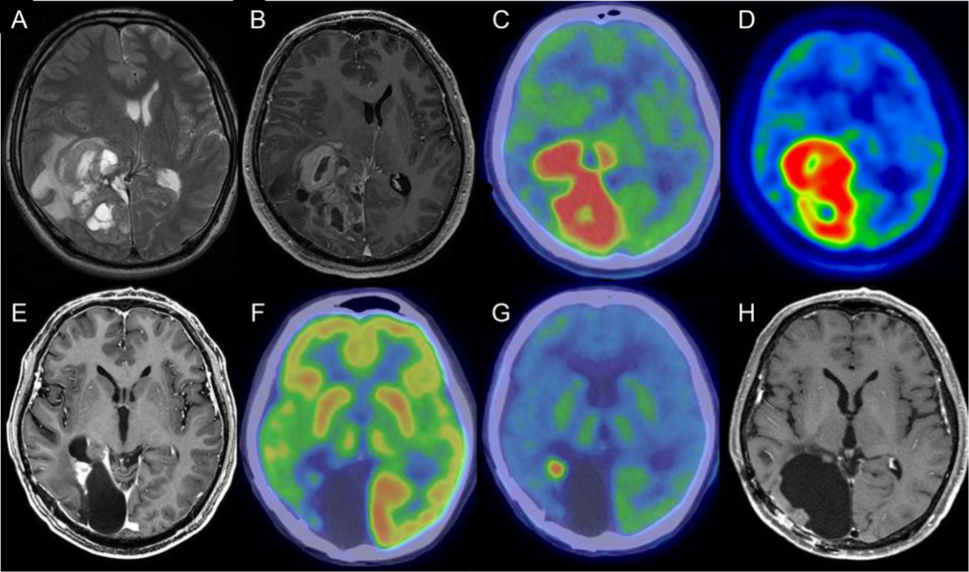
**Magnetic resonance imaging (MRI) and positron emission tomography (PET). (A)** T2-weighted image showing a huge multicystic mass in the right occipitoparietal area with marked surrounding edema and shift of the midline structures to the left side. **(B)** Each cyst wall and adjacent cortical mass was enhanced with contrast medium. **(C)** Fluorodeoxyglucose (FDG) PET showing high accumulation in the right occipitoparietal area. **(D)** Methionine (MET) PET showing high accumulation in the right occipitoparietal area. **(E, F)** MRI and PET findings at the time of recurrence. **(E)** Small enhanced mass adjacent to the cavity formed by removal of the tumor. (F) FDG-PET showing no accumulation in the mass. **(G)** MET-PET showing high accumulation in the mass. **(H)** MRI 4 years after the first operation.

A 49-year-old man was admitted to the hospital complaining of headache, vomiting, and mild left hemiparesis. Magnetic resonance imaging (MRI) showed a huge multicystic mass in the right occipitoparietal area with marked surrounding edema and a shift of the midline structures to the left side (Figure 1A). The cyst wall and adjacent cortical mass were enhanced with contrast medium (Figure 1B). 18F-Fluorodeoxyglucose (FDG) and methionine (MET) positron emission tomography (PET) revealed high accumulation in the right occipitoparietal area (Figure 1C, D).

Near-total resection of the tumor was performed. After glioblastoma (GBM) was pathologically diagnosed, the patient had local radiation using tomotherapy (60 Gy/30 fractions), with concomitant chemotherapy consisting of temozolomide (75 mg/m^2^/day). After a 4-week break, the patient received 19 cycles of adjuvant temozolomide (150 mg/m^2^/day) for 5 days every 28 days. A small contrast-enhancing lesion was seen on MRI close to an extraction cavity 22 months after the first operation. Because MET-PET showed a high accumulation in the mass, although none was detected using FDG-PET (Figure 1F, G), a second operation was performed, and the recurrence of GBM was diagnosed. Furthermore, the patient continues to receive 31 cycles of adjuvant temozolomide (200 mg/m^2^/day) for 5 days every 28 days and is living without recurrence 4 years after the first operation (Figure 1H).

### Pathological findings

**Figure 2:**
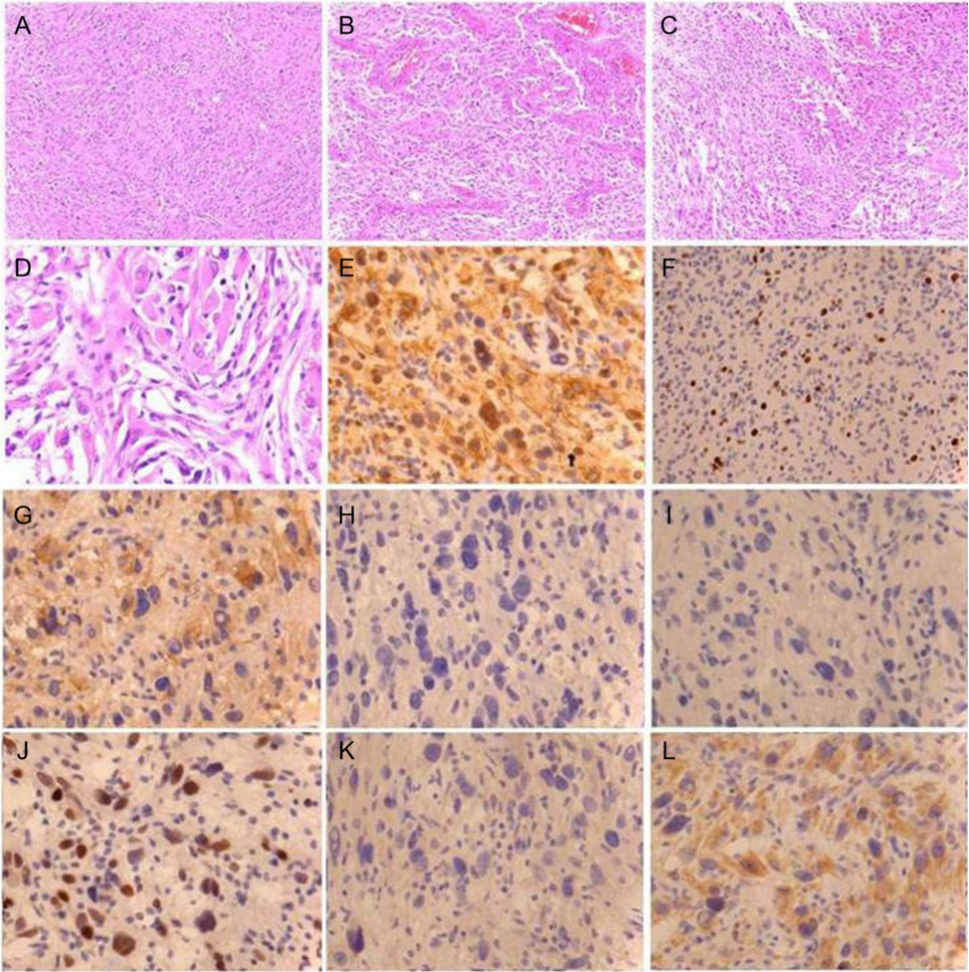
**Histopathological features of the** ***BRAF*** **V600E-positive glioblastoma. (A-D)** Hematoxylin and eosin **(H & E)** staining. **(A, D)** Multiple atypical spindle cells mixed with gemistocytes. **(B)** Microvascular proliferation, **(C)** pseudopalisading, **(E)** GFAP positive, **(F)** Ki67 (index was approximately 10%), **(G)** EMA positive, **(H)** pan-cytokeratin (AE1/AE3) negative, **(I)** EGFR negative, **(J)** P53 positive, **(K)** IDH1 negative, and **(L)** *BRAF* V600E positive.

Numerous atypical spindle cells were interspersed with gemistocytes (Figure 2A, D), and microvascular proliferation and pseudopalisading were present (Figure 2B, C). Tumor cells were highly positive for glial fibrillary acidic protein (GFAP; Figure 2E), and the Ki67 index was approximately 10% (Figure 2F). Expression of cytokeratins was undetectable in EMA^+^ tumor cells (Figure 2G, H). Findings of tumor cells negative for epidermal growth factor receptor (EGFR) but positive for P53 are typical of secondary GBM (Figure 2I, J). Expression of the *IDH1* R132H mutant or the *IDH1* R132H mutation was not detected using immunohistochemistry or NGS analysis, respectively (Figure 2K). In contrast, expression of the *BRAF* V600E mutant was detected using immunohistochemistry, and the *BRAF* V600E mutation was detected using NGS (Figure 2L).

### DNA extraction and NGS

DNA was extracted from formalin-fixed paraffin-embedded (FFPE) sections using a NucleoSpin DNA FFPE XS Kit (Macherey-Nagel, Düren, Germany), and 225 ng of each genomic DNA sample was subjected to target amplification and library preparation for NGS analysis using a HaloPlex Cancer Research Panel (ABL1, JAK2, AKT1, JAK3, ALK, KIT, AR KRAS, ATM, MAP2K1, BRAF, MAP2K4, CDKN2A, MET, CSF1R, NOTCH1, CTNNB1, NPM1, EGFR, NRAS, ERBB2, PDGFRA, ERBB4, PIK3CA, FANCA, PIK3R1, FANCC, PTEN, FANCF, RET, FANCG, RUNX1, FGFR1, SMAD4, FGFR2, SMO, FGFR3, SRC, FLT3, STK11, HRAS, TP53, IDH1, VHL, IDH2, WT1, MAP2K2; Agilent Technologies, Santa Clara, CA, USA) according to the manufacturer’s instructions. The target enrichment library pool was sequenced using a MiSeq (Illumina, San Diego, CA, USA). The sequence data were aligned, analyzed, and visualized using SureCall 2.0 software (Agilent Technologies).

### Immunohistochemistry

Blocks of FFPE tumor sections were cut to a thickness of 4 μm, deparaffinized, and treated with an antigen unmasking solution (Immunosaver, Nissin EM Co. Ltd., Tokyo, Japan) at 90°C for 45 min and incubated with blocking solution (3% H_2_O_2_) at room temperature. The anti-BRAF V600E (VE1) antibody (Ventana Medical Systems, Tucson, AZ, USA) was diluted 1:2,000 and incubated with sections for 16 h at 4°C. Antibody-antigen reactions were detected using Bond Polymer Refine Detection reagents (Leica Biosystems, St. Louis, MO, USA). Additional sections were incubated for 1 h at room temperature in phosphate-buffered saline (PBS) with the antibodies as follows: ready-to-use formats of anti-GFAP clone GA5, anti-Ki67 clone SP6, anti-EMA clone E29, anti-pan-cytokeratin clone AE1/AE3, and anti-EGFR clone 31G7 (all from Nichirei Bioscience, Tokyo, Japan); anti-P53 antibody diluted 1:100 (Dako, Glostrup, Denmark); or an anti-isocitrate dehydrogenase 1 (IDH1) R132H antibody clone H09 diluted 1:50 (Dianova, Hamburg, Germany). After incubation with primary antibodies, the sections were reacted with a peroxidase-conjugated secondary antibody (catalog number 424154, Nichirei Bioscience) for 1 h at room temperature and rinsed with PBS. The Histofine DAB kit (Nichirei Bioscience) was used to detect antigen-antibody complexes.

## Conclusions

The *BRAF* V600E mutation may occur at low frequency in adult c-GBM. This mutation was detected by the direct sequence method of Sanger method or immunohistochemistry. NGS techniques are in wide use because mutations are detected with greater sensitivity compared with Sanger sequencing [7,8]. Therefore, we have performed NGS analysis to detect *BRAF* mutations in tissues of 13 patients with c-GBM (11 primary and 2 secondary cases) who were treated at Hokuto Hospital. However, the *BRAF* V600E mutation was detected in only one case (7.7%; Table 1). The validity of the data is supported by the detection of *BRAF* V600E in the tumor tissue of the same patient after the tumor recurred.

**Table 1:** **Result of next-generation sequencing analysis of glioblastoma**

**Case**	**Age**	**Gender**	**Type**	**Gene**	**ID**	**Depth**	**Codon**	**Amino acid**
1	39	M	Primary	FANCA	rs2239359	264	Ggc/Agc	G501S
				STK11	rs59912467	157	ttC/ttG	F354L
2	49	M	Primary	PDGFRA		458	cCg/cGg	p553R
				BRAF	rs121913227	311	gTg/gAg	V600E
				FANCA	rs2239359	1,141	Ggc/Agc	G501S
Recurrence				PDGFRA		458	cCg/cGg	p553R
				BRAF	rs121913227	311	gTg/gAg	V600E
				FANCA	rs2239359	1,141	Ggc/Agc	G501S
3	60	M	Primary	VHL	rs369018004	8,156	Ctg/Gtg	L129V
				FANCA	rs2239359	14,888	Ggc/Agc	G501S
4	42	F	Secondary	FANCA	rs2239359	9,366	Ggc/Agc	G501S
5	55	M	Secondary	IDH1	rs121913500	4,571	cGt/cAt	R132H
				ATM		14,928	Ggt/Agt	G2695S
				FANCA	rs2239359	18,284	Ggc/Agc	G501S
6	79	M	Primary	FANCA	rs2239359	18,284	Ggc/Agc	G501S
7	80	M	Primary	FANCA	rs2239359	3,313	Ggc/Agc	G501S
				STK11	rs59912467	2,723	ttC/ttG	F354L
8	80	M	Primary	FANCA	rs2239359	18,161	Ggc/Agc	G501S
9	58	M	Primary	FANCA	rs2239359	18,161	Ggc/Agc	G501S
				STK11	rs59912467	2,723	ttC/ttG	F354L
10	62	M	Primary	FANCA	rs2239359	513	Ggc/Agc	G501S
11	66	M	Primary	FANCA	rs2239359	8,387	Ggc/Agc	G501S
12	63	F	Primary	PDGFRA	rs2228230	2,959	gtC/gtT	V824
				FANCA	rs2239359	7,658	Ggc/Agc	G501S
13	62	M	Primary	FANCA	rs2239359	8,036	Ggc/Agc	G501S

When we reviewed the pathology of this case, the tumor was not an epithelioid or a giant cell GBM because foci with glandular and ribbon-like epithelial structures and multinucleated giant cells were not present. Classical GBM was confirmed by two expert neuropathologists (H.N. and S.T).

Although there is no detailed report of a *BRAF* V600E-positive adult c-GBM, to our knowledge, there is a study of two such cases [9]. The tumors of both patients were located within the right parietal lobe. Interestingly, the tumor of our present patient was located in the right occipitoparietal lobe. Moreover, our patient with BRAF V600E-positive adult c-GBM was alive when this manuscript was submitted, 4 years after the first operation. In contrast, the patients studied by Chi et al. [9] survived for 19 and 36 months. The *IDH1* mutation serves as a good prognostic factor for patients with GBM but was not detectable using NGS or immunohistochemical analyses. These data are consistent with the results of a study of two patients with *BRAF* V600E-positive c-GBM [9].

Although the number of patients was small, these three patients with *BRAF* V600E-positive GBM survived relatively longer compared with patients without this mutation. Therefore, tumors with the *BRAF* V600E mutation may represent a more favorable subtype of GBM. More patients with GBM must be analyzed to conclude that the *BRAF* V600E mutation is a potential prognostic marker for GBM. Moreover, inhibitors of B-Raf protein kinase activity may serve as efficacious drugs for treating patients with *BRAF* V600E-positive GBM [10,11]. Therefore, we suggest that we should perform routine genetic testing of *BRAF* V600E mutation, which might provide effective alternatives to treat patients with GBM.

## Consent

Written informed consent was obtained from the patient for publication of this case report and any accompanying images. A copy of the written consent is available for review by the Editor-in-Chief of this journal.
